# Malaysian *Carica papaya* L. var. Eksotika: Current Research Strategies Fronting Challenges

**DOI:** 10.3389/fpls.2018.01380

**Published:** 2018-09-18

**Authors:** Rogayah Sekeli, Muhammad Hanam Hamid, Roslinda A. Razak, Chien-Yeong Wee, Janna Ong-Abdullah

**Affiliations:** ^1^Biotechnology and Nanotechnology Research Centre, Malaysian Agricultural Research and Development Institute, Serdang, Malaysia; ^2^Department of Cell and Molecular Biology, Faculty of Biotechnology and Biomolecular Science, Universiti Putra Malaysia, Serdang, Malaysia

**Keywords:** *Carica papaya* var. Eksotika, dieback disease, shelf-life, -omics technologies, genetic engineering

## Abstract

*Carica papaya* L. or commonly known as papaya, is a major tropical crop consumed worldwide either as a vegetable or fresh fruit or processed products. In Malaysia, papaya was initially planted as a smallholder crop throughout the country. Eventually after 15 years of breeding and selection, a new variety, named *C. papaya* L. var. Eksotika, was released by the Malaysian Agricultural Research and Development Institute (MARDI) in 1987. This event changed the outlook of papaya planting from a smallholder crop to a plantation crop. Despite the blooming papaya business, the industry faced various disease issues that jeopardize its future. The most devastating was the papaya dieback disease, which affected approximately 800 hectares of plantation, destroyed approximately 1 million trees nationwide with total losses estimated at US$ 58 million. Even though Eksotika is a favored commercial variety with good eating and aesthetic quality fruit, its potential for more lucrative distant markets is tarnished with its short-shelf life fruits. Several strategies had been reported to address the challenges faced by Eksotika specifically against the dieback disease and the fruit’s short shelf-life. This review focuses on *C. papaya* L. var. Eksotika particularly on the strategies to address the challenges faced in order to sustain the economic value of this crop plant, which had contributed significantly to the Malaysian economy.

## Introduction

*Carica papaya* L., commonly known as papaya, papaw or pawpaw (in Australia), and Mamao (in Brazil), is one of the major tropical crops consumed worldwide either as a vegetable or fresh fruit or processed products like jam, preserved and canned. Generally, the papaya tree is fast-growing, semi-woody, produces latex, and usually short-lived. The white latex of papaya containing the enzyme, papain, can be used to tenderize meat. Papaya cultivars are usually distinguish based on the number of leaf main veins, types of stomata, color of the leaf petiole, number of lobes at the leaf margins, leaf shape, and structure of the wax on the leaf surface (da Silva et al., 2007). It is a dicotyledonous plant with a somatic chromosome number of 18, and belongs to the family *Caricaceae* comprising of four genera namely *Carica, Jacaratia, Jarilla*, and *Cylicomorpha*. Nakasone and Paull (1998) reported that *Carica, Jacaratia*, and *Jarilla* were from tropical America, while *Cylicomorpha* had its origin in equatorial Africa. However, earlier report by Purseglove (1968) commented that papaya plant was originated from the Caribbean coast of Central America via natural hybridization between *Carica peltata* and another wild species of papaya. In fact, papaya was found to be distributed from Mexico to the Panama (Nakasone and Paull, 1998), and spread to the Caribbean and Philippines during the exploration of the Spanish explorer, Don Francisco Marine, in the 16th century, and eventually became an export crop in 1948 (Fitch, 2005).

Interestingly, the papaya seeds from the Philippines were distributed in 1550 to Malacca (a state in Malaysia) and also to India. Since then, papaya is planted for subsistence consumption and commercial purposes. As such, global production of papaya rose gradually and achieved over 13 million metric tons in 2016 (FAOSTAT, 2017). Nevertheless, the papaya industry, as a whole, has its share of the good and bad times. In Malaysia, the rise of papaya as a major export crop is a challenging but rewarding journey. This review focuses specifically on the commercially sought after *C. papaya* L. var. Eksotika, from how it was derived, the challenges it faced, the used of transformation technology to enhance the quality of the fruits and plant, and potential -omics strategies to address ongoing challenges in order to sustain the economic value of this crop plant.

## How Eksotika Began

**FIGURE 1:**
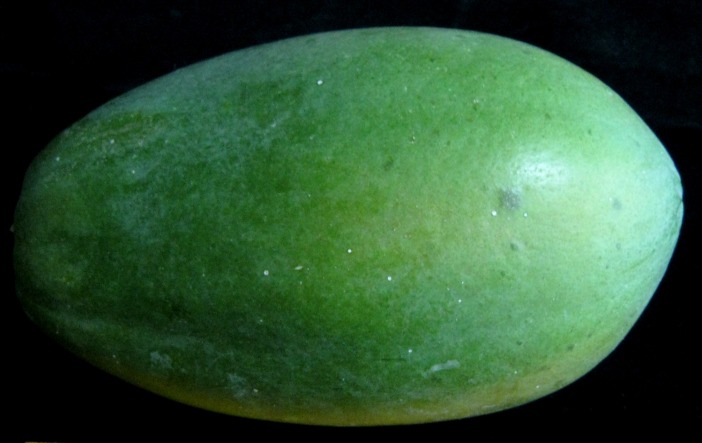
Fruit of *Carica papaya* L. var. Eksotika.

In 1972, the papaya scenario changed when the Malaysian Agricultural Research and Development Institute (MARDI) started a backcross breeding program using the papaya variety, Sunrise Solo, from Hawaii. Sunrise Solo has excellent eating qualities but possesses poor yield and small fruit size (Glenn, 2016). It was crossed with the locally adapted, large fruited Subang 6 (Chan, 1987). Subsequent progenies underwent a series of “self-pollination” and backcrossing to Sunrise Solo to re-constitute its excellent eating qualities while selecting for larger fruit size of the Subang 6. After 15 years of breeding and selection, a line called Backcross Solo with the features of the original Sunrise Solo and having increased fruit size and local adaptability of Subang 6, was finally selected. In 1987, this new variety, named as Eksotika papaya (**Figure 1**), was released by MARDI. It has robust growth characteristics with ability to grow in a wide range of soil types with good drainage system. In addition, Eksotika is a good bearer, which can achieve fruit production of about 60 tons per harvested area per year. The Eksotika fruit is small to medium size weighing about 400–800 g. The fruit has orange-red flesh with pleasant aroma and high sugar content (13–15°Brix). These attractive characteristics of high yield and high aesthetic values allowed it to become the main variety grown for commercial and export purposes. It was highly recommended for the export markets like China, Hong Kong, Singapore, the Middle East, and Europe (Sew et al., 2011).

The release of Eksotika enabled the papaya plantation to become one of the potential industries in Malaysia contributing to world export trading averaging more than 37 thousand tons of fruits annually from years 1993 to 2013; and ranked as the second biggest contributor of world papaya with an export value of about RM100-120 million per year (FAOSTAT, 2017). Before the advent of Malaysia’s Eksotika papaya variety, the existing papaya varieties were inconsistent in yield and generally the fruits had poor eating qualities. Nevertheless, the popular varieties were limited to Setiawan, Batu Arang, and Subang 6. These varieties have large fruit size, which is inconvenient for handling and serving.

## The Challenges of Eksotika

Like other crops in Malaysia, papaya industry faces various issues that jeopardize its future. There are numerous diseases and pests affecting papaya production, other than substandard quality fruits to be marketed inside or outside the country.

### Eksotika Diseases

A wide range of diseases had been documented for papaya varieties, whereby majority were due to pests (fruit flies, spider mite, and nematodes) aiming at the papaya foliage, fruits, and roots (Pantoja et al., 2002). Besides, microorganisms-related diseases were also reported such as papaya fruit spot (Chupp, 1953), papaya leaf curl (Nariana, 1956), papaya mosaic (Conover, 1962), papaya powdery mildew (Alcorn, 1968), papaya ring spot (Cook and Milbrath, 1971), fungal root rot (Ko, 1971), and a later disease is the bacterial dieback or more commonly known as papaya dieback disease (Maktar et al., 2008).

**FIGURE 2:**
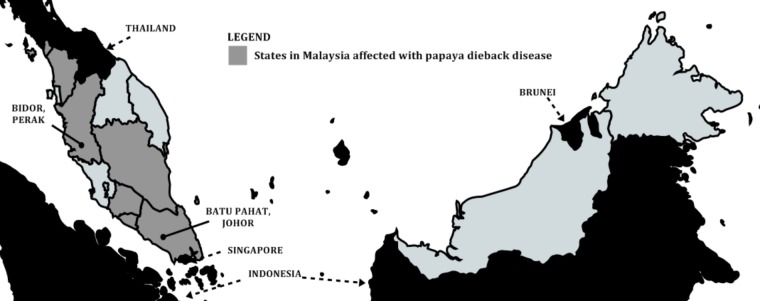
Outbreaks of papaya dieback disease across Malaysia. States affected by the disease from 2003 to 2006. Johor was the first affected state, followed by Perak and other states.

Outbreak of papaya dieback disease was first identified in late 2003 near Batu Pahat, Johor (a state in southern Malaysian peninsula close-by Singapore) by the Johor State Department of Agriculture. Another devastating incident was later reported in Bidor, Perak (a state in the northern part of Malaysian peninsula close-by Thailand) in October 2004. By the end of 2006, the disease had spread to five other states on the west coast of the Malaysian peninsula namely Melaka, Negeri Sembilan, Pahang, Kedah, and Perlis (**Figure 2**). The outbreak affected ca. 800 hectare and resulted in the destruction of approximately 1 million trees nationwide. The damaged trees caused total fruit yield losses with estimated 200,000 metric tons, equivalent to US$ 58 million (Maktar et al., 2008; Roshidi, 2010). Besides Eksotika, other varieties affected included Solo, Hong Kong, and Sekaki.

FAOSTAT (2017) reported that papaya fruit production in Malaysia was reduced nearly 40% while the export value declined up to 70% from 2003 to 2011. Papaya dieback disease was a significant reason contributing to these upsetting figures. At the beginning, there was ambiguity on the identity of the causal agent for this disease. Although it was first reported as *Erwinia papayae* by Maktar et al. (2008), later Mat Amin et al. (2011) from MARDI confirmed the causal agent of papaya dieback disease in western parts of Malaysian peninsula was an *Erwinia* genus from the *mallotivora* species. Both species of this Enterobacteriaceae family exhibited similar symptoms but isolates from infected papaya trees unveiled distinguishable biochemical tests positive for *Erwinia mallotivora* rather than to *E. papayae.* This was further supported by earlier observations made by Gardan et al. (2004) who reported that *E. papayae* was said to cause canker, however, in the advanced stage of papaya dieback as reported by Maktar et al. (2008), there was no canker symptom observed.

**FIGURE 3:**
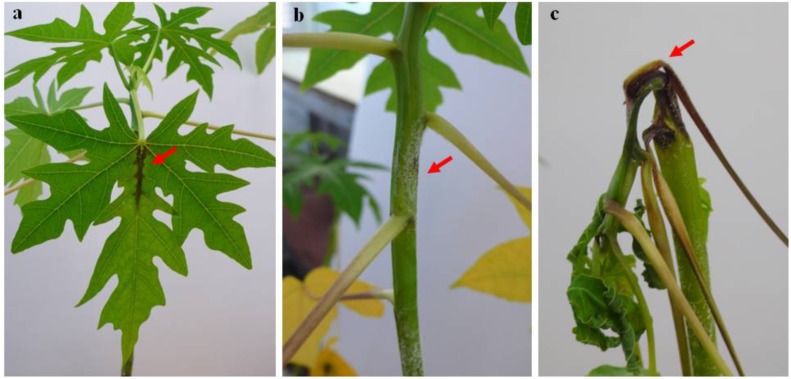
Papaya dieback disease symptoms development on papaya seedlings artificially infected with the pathogen, *Erwinia mallotivora*. **(a)** Necrotic lesion developed along the vein of infected leaf, **(b)** water soaked lesion developed on infected stem, and **(c)** shrunken and bending of infected stem, leading to ultimate plant death. Arrows indicate the affected regions.

The mode of action of the *Erwinia* pathogen is through invading and colonizing the entire parts of a papaya plant including the shoot, leaf, bark, and fruit. Early symptoms of papaya dieback disease include yellowing and necrosis along leaf edges (**Figure 3a**) followed by water-soaked areas on the bases of leaf stalks, crowns and along the leaf mid-ribs. Subsequently, necrotic and water-soaked areas developed on stems (**Figure 3b**) and spread to the internal tissues, followed by secondary fungal infections associated with the septic parts (Maktar et al., 2008). In some cases, blackened and greasy spot on infection points were observed (Mat Amin et al., 2011). Progressive infection on the crown resulted in total disruption of the meristematic part of the papaya tree that bears the fruit. In the last infection stage, bending of water-soaked leaf stalks (**Figure 3c**) occurred leading to “die back” (aptly described the name of the disease), and the ultimate death of the tree. Until now, there is no effective way to control the disease once the pathogen gained entry into the plant.

### Post-harvest Losses

Another critical issue faced by the Malaysian papaya industry causing major postharvest losses is the short shelf-life of the fruit. The Eksotika papaya has poor keeping quality and is unable to withstand long distant markets due to its fast ripening attribute. Such attribute is considered unfortunate since the fruits need to be distributed and reach the consumer in a short period of time. The short shelf-life of the papaya fruit contributes to poor appearance, texture, flavor and overall quality, and can lead to postharvest losses up to 30–40% if the fruit is not consumed in time.

In general, the papaya fruits are harvested at the pre-climacteric stage, Index 2 (with traces of yellow), and allowed to ripen by keeping for 4–6 days under ambient temperature (25–28°C). Nevertheless, the storage-life could be increased to 14–21 days when stored at 10–12°C under modified atmosphere (Rohani et al., 1993). However, increasing the storage-life to 3 weeks is still insufficient to meet the duration for distant markets. In addition, the fruit is sensitive to low temperatures, which cause chilling injury and rapid deterioration of the fruit quality. Hence, the fruit’s shelf-life cannot be further increased by storing at temperature lower than the recommended 10°C during transportation. Inversely, if the fruits were harvested at a more advanced stage they would ripen rapidly due to ethylene production, and this might result in over ripening and subsequent postharvest losses. Thus, proper harvesting time, method for harvesting, type of packaging and storing conditions are very important factors to reduce such postharvest losses. A right temperature and also precise humidity are very crucial, and are determined for each market location to avoid postharvest losses. Both characteristics of short shelf-life and sensitivity to low temperatures need to be improved to enhance its export potential in reaching long distant markets and at a cheaper rate by using sea transport. Numerous attempts had been made through conventional breeding and manipulation of postharvest approaches but the issues were yet to be solved successfully.

## Strategies to Address the Challenges Faced by Eksotika

Eksotika had faced many challenges, and will continue to do so for as long as it is economically viable. Concerted efforts based on short-, mid-, and long-term strategies had been attended to address the challenges faced by Eksotika specifically against the dieback disease and the fruit’s short shelf-life.

## Short- and Mid-Term Strategies

The uses of pesticides or antibiotics were found ineffective in controlling papaya dieback disease. Plants that exhibited disease symptoms had to be destroyed immediately as recommended under the Plant Protection Act 1974. Currently, early detection of the disease symptoms and destroying the affected plants seemed to be the best control strategy in preventing outbreaks spreading into other areas. This is because the disease is not soil-borne, and therefore, it is possible to control the spread of the disease by careful removal of infected plants or leaves to prevent further damages and infection of surrounding healthy plants. As recommended by breeders, replanting in infected areas after removal of the infected plants were done only after 6 months to avoid infection of newly cultivated plants with the same disease. However, this is a short-term strategy and is ineffective in the long run with the papaya plantation still under threat and the issue of dieback remains unsolved.

The search for an alternative strategy to cope and overcome the disease efficiently is urgently needed. Researchers had begun to turn their attention to plant innate immunity to combat pathogen attack such as the plant Systemic Acquired Resistance (SAR) mechanism and application of microbial consortium to enhance papaya resistance against dieback. Plants upon infection were said to be capable of developing SAR, which induced the defense mechanism and conferred long lasting protection against a spectrum of microorganisms (Conrath, 2006). Hence, it was hypothesized that healthy plants subjected to external application of microbial proteins or chemical inducers were able to activate their defense genes or proteins and fight off infections. Based on this foundation, selected recombinant proteins from *E. mallotivora* and chemicals had been tested to assess their roles in papaya plant defense against the dieback (Abu Bakar et al., 2016). Attempts had also been made to investigate bacteria cell-to-cell communication system known as quorum sensing and its signal molecule, *N*-Acyl-L-Homoserine Lactone (AHL), which is responsible for pathogenicity in some Gram-negative plant pathogens. In contrast, quorum quenching enzyme, *N*-acyl-L-homoserine lactonase (Ahl-lactonase) and *N*-acyl-homoserine lactone acylase (Ahl-acylase) which are able to degrade AHLs and eliminate pathogen virulence (Wang et al., 2004; Sio et al., 2006). Ahl-lactonase was discovered in six soil rhizosphere bacteria, which were said to have potentials for use as biological control agents against the papaya dieback agent (Mat Amin et al., 2016a,b). Recent findings by Krishnen (2017) indicated that application of microbial consortium on the papaya seedlings could be used to control the spread of the disease. The said consortium comprised of different microbial cultures possessing antibacterial properties that conferred resistance to germinated seeds against the papaya dieback disease. The questions that came to mind are how effective is this method and is it sustainable? What about cost effectiveness in terms of field planting for this crop plant?

So far there is only one reported work on short-term strategy to address the fruit’s short shelf-life challenge. Rashid et al. (2015) revealed that low-dose gamma irradiation following hot water immersion of papaya fruits helped to control postharvest fungal infection and extended the fruit’s shelf-life up to 28 days with good commercial quality. However, this still does not address the problem associated with the fruit’s inherent short shelf-life property.

## Long-Term Strategy

### Genetic Engineering

Modern biotechnology provides a cutting-edge technology in many areas such as agriculture, medicine, and environment. Genetic engineering is one of the applications of modern biotechnology that has been widely used for improvement of crop quality such as increased yield, resistant to pests and diseases, and improved nutritional value.

The dieback disease has caused a major hiccup in the Malaysian papaya industry. Current genetic engineering research on papaya is focused on developing Eksotika papaya resistant to dieback disease. In 2013, screening for potential genes involved in quorum quenching to be used in genetic manipulation studies against the dieback disease was initiated (Mat Amin et al., 2013). Strategically, a molecular technique to disrupt the quorum-sensing signaling of *E. mallotivora* was identified with the hypothesis that it was possible to prevent the infection and survival of *E. mallotivora* in the papaya plant. Growth of *E. mallotivora* can be arrested and consequently delays or inhibits the development of dieback disease. Ahl-lactonase is an enzyme capable of inactivating the AHL molecules that is crucial for bacterial communication or quorum sensing. The inactivation mechanism is via hydrolysis of the lactone bond of AHL molecules (Dong et al., 2001). It was postulated that transgenic papaya plants expressing *Ahl-lactonase* are capable of quenching the pathogen quorum-sensing signaling, and enhance their defense against dieback disease.

Mat Amin et al. (2013) successfully isolated and characterized two potential genes, *acyl-homoserine lactonase* SP24 and *acyl-homoserine lactonase* CHB18, which have anti-pathogenic activity. The antimicrobial activities of both enzymes were validated *in vitro*. Sekeli et al. (2015) transformed those genes into embryogenic callus of Eksotika papaya by using *Agrobacterium*-mediated transformation method. The transgenic plants produced were challenged with *E. mallotivora* in a controlled and contained environment to evaluate the degree of resistance. The potential transgenic T_0_ papaya line, which showed tolerance to dieback was successfully identified. However, contained field trials are still ongoing to confirm their resistance against the dieback disease.

Besides papaya dieback, MARDI’s researchers had been diligently pursuing the development of a new variety of Eksotika papaya bearing fruits with longer shelf-life. In 1990, MARDI started the research on delayed ripening papaya with the successful development of a tissue culture regeneration and transformation system, and identification of *1-aminocyclopropane-1-carboxylic acid oxidase* 2 (*ACO*2) gene associated with ripening. Following which, transgenic and silencing technologies were used to develop new Eksotika papaya variety with delayed fruit ripening trait by manipulating the *ACO*2 gene in the pathway (Sekeli et al., 2013). The research findings showed ethylene production was greatly reduced by manipulating *ACO*2 gene using antisense technique. Physical stature of the transgenic plants was not significantly different from the non-transformed seed-derived papaya plants. Physiological evaluations of the transgenic fruits showed up to 15 days delayed in ripening compared with 4 days of the non-transformed seed-derived papaya fruits. The total soluble solid (TSS) of the transgenic fruits was comparable to the non-transformed seed-derived fruits with similar profile (11–15°Brix), implying the transgenes did not affect the TSS content. The transgenic fruits remained firm for additional 4–8 days at room temperature (25 ± 2°C) after achieving the full maturity index (Index 6), whereas the non-transformed seed-derived fruits lost their firmness after 2 days. This study proved that reduction of ethylene production in Eksotika papaya by manipulating the *ACO*2 gene using antisense technique successfully delayed the ripening and increased the fruit shelf-life (Sekeli, 2014).

### Functional Genomics

It is crucial to elucidate the mechanism of a host-pathogen interaction and responses at molecular level before a useful strategy can be devised and implemented. Rapid progress and change of functional genomics analyses had allowed new insights and challenges in understanding the complex mechanism of plant disease resistance (Rosli and Martin, 2015; Seo and Choi, 2015; Singh et al., 2017). Functional genomics research focuses on the dynamic aspects involving gene expression and protein interactions by using high-throughput -omics technologies, in contrast to the single gene approach of classical molecular biology techniques, which study DNA sequence and structure (Bunnik and Le Roch, 2013).

The first reported study of papaya dieback disease using functional genomics approach was in 2010. At the beginning, research was mainly focused on identification of plant defense genes. Host gene expression study by initiating host-pathogen interaction to induce papaya dieback was conducted using the serial analysis of gene expression (SAGE) technology (Khairun et al., 2010). Specific causal agent of papaya dieback disease was yet to be identified at that time and a pathogen cocktail was used to induce the disease. Gene expression profiles and a list of genes in short tag sequences, which showed transcriptional changes due to the infection, were obtained from the study. Subsequently, a potential defense-related gene, *zinc finger protein*, was selected for further characterization (Wee et al., 2011, 2014b; Zaidan et al., 2011).

It took years to identify the actual causal agent of papaya dieback disease in Malaysia. In 2011, it was reported a new pathogen of papaya, *E. mallotivora*, was the causal agent for the papaya dieback disease in West Malaysia (Mat Amin et al., 2011). This identification had a marked influence on subsequent work as it changed the scenario on how to tackle the disease problem faced, which started off with attempts to understand the pathogenic mechanism. Thereafter, efforts were targeted at sequencing the genome of the pathogen (Redzuan et al., 2014). Several putative virulence genes and proteins were identified, followed by gene expression profiles validation using real-time PCR (Badrun et al., 2016) and quantitative proteomics validation (Abu Bakar et al., 2017). Genome studies on the pathogen indirectly provided important information on the host–pathogen interactions for the dieback infection in affected papaya plants. They set the stage and opened up further directions for future researches including comprehensive investigation on the pathogenicity genes, identification of potential pathogen-inducible and defense-related genes in host plant, discovering papaya miRNA for enhancing host defense mechanism, and generating transgenic resistant papaya through disruption of pathogen virulence factors and expression of antimicrobial proteins of non-plant origin in papaya (Bunawan and Baharum, 2015).

### Other -Omics

Transcriptomics, proteomics and bioinformatics are crucial and supportive of the genomics data to elucidate the complex mechanism of plant–pathogen interaction. Ling et al. (2016) recently reported the transcriptome profile of Eksotika (susceptible variety) in comparison to Viorica (tolerant variety; Sarip et al., 2015) papaya. Frontier technology, RNA-seq, was embarked to identify potential defense-related genes expressed differentially in plantlets of both varieties artificially infected with *E. mallotivora*. Potential novel Long Intervening Non-coding RNAs (lincRNAs) were also discovered from the high-throughput transcriptomic sequences obtained, and further *in silico* functional analyses revealed their potential roles in plant defense responses (Rozano et al., 2015, 2016).

The use of bioinformatics tool has enabled the integration of current huge papaya transcriptome sequencing data and the papaya genome data published in 2008 to predict a more holistic view of the disease mechanism in papaya (Abdullah-Zawawi et al., 2015; Rozano and Abdullah-Zawawi, 2017). The information gathered on the host–pathogen interactions during infection is helpful since it can be structured using a software network analysis to facilitate strategy design in addressing the disease issue. Furthermore, identification of potential papaya genes related to defense toward pathogen in vascular bundle were also made possible using data mined from the public domain, *the Arabidopsis Information Resource* (TAIR) database^1^, *the Institute for Genomic Research* (TIGR) *Plant Transcript Assemblies* (TA) database^2^, *the Kyoto Encyclopedia of Genes and Genomes* (KEGG) pathway ^3^ and Phytozome database version 10.3 ^4^. Four potential papaya vascular-related defense genes, *glycerol kinase* (*NHO*1) (GenBank Accession No. KT833845), *mitogen-activated protein kinase* 4 (*MPK*4) (GenBank Accession No. KT833849), *EF-TU receptor* (*EFR*) (GenBank Accession No. KT833848), and *pathogenesis-related protein* 1 (*PR*1) (GenBank Accession No. KT833847) were selected and characterized (Hamid et al., 2014; Wee et al., 2014a; Hamid, 2017). Since plant defense mechanism functions in a complicated network of multiple genes interactions, bioinformatics tools made it easy to investigate the papaya mitogen-activated protein kinase (MAP kinase) *MPK4/mitogen-activated protein kinase kinase kinase* 1 (*MEKK1*)*/mitogen-activated protein kinase kinase* (*MKK*) genes interactions to predict their localizations and respective signature domains for their basic functional roles (Hamid et al., 2017).

Tobacco, tomato and *Arabidopsis* are commonly used as model systems in validating gene function. Several papaya defense-related genes had been successfully over-expressed in tobacco plant model system and quantitative real-time PCR demonstrated high expression for all genes tested in the model plant (Zaidan et al., 2012). However, these systems may not be suitable for validating defense-related genes in response to papaya dieback disease since they are not the hosts for the dieback causal pathogen. Furthermore, these model systems may not confer accurate, precise, and actual results when used to validate the genes functions of a more complex crop such as papaya. Therefore, a papaya model system was developed to enable a rapid and high-throughput gene function validation method for functionality test of novel genes identified from in-house developed papaya database. Such effective papaya model system for gene over-expression study was successfully developed by Wee and Hamid (2014). The gene of interest was cloned into a virus vector, pEAQ (Sainsbury et al., 2009), and the construct was transformed into the *Agrobacterium*. Over-expression of the gene was conducted by infiltrating the *Agrobacterium* into papaya plant. Hamid (2017) over-expressed the potential papaya vascular-related defense genes, *glycerol kinase* (*NHO1*) (GenBank Accession No. KT833845), *mitogen-activated protein kinase* 4 (*MPK*4) (GenBank Accession No. KT833849), *EF-TU receptor* (*EFR*) (GenBank Accession No. KT833848), and *pathogenesis-related protein* 1 (*PR*1) (GenBank Accession No. KT833847), in the said papaya model system followed by artificially infecting the infiltrated plants with *E. mallotivora*. Phenotypic changes and disease symptoms development were observed, and expression levels of these genes were affected by the pathogen infection when analyzed using real-time PCR.

Proteomics studies were also embarked to gather more information on host–pathogen interactions at the protein level, which may close the gap in illustrating specific gene function in a biological system. A comparative proteomics analysis was used to investigate molecular response of tolerant and non-tolerant papaya plants to dieback disease (Supian et al., 2016). In coupling with bioinformatics analysis, five differentially expressed proteins, cysteine protease, phosphoribulokinase, leucine aminopeptidase 3, peptidyl-prolyl *cis*-trans isomerase CYP20-2 and PRH26 protein, were annotated as defense and stress response proteins. It was hypothesized that early defense responses of papaya toward pathogen infection were mediated by antioxidant system. Thus, early defense responses of the susceptible papaya cultivar toward the pathogen infection was examined by measuring superoxide dismutase (SOD) and peroxidase (POD) enzymatic activities. Infected leaf proteome was also analyzed to acquire insights of the global cellular responses (Supian et al., 2017). Noted were enhanced activities of the antioxidant enzymes and elevation of proteins involved in energy production and stress response indicating the initiation of early defense response once the plant was infected.

## Concluding Remarks and Future Outlooks

We are in the era of a technological revolution that is transforming our lifestyles at an exponential pace, transcending the boundaries that separate the physical, digital, and biological spheres. The existence of cyber infrastructure, big data management and data mining capabilities warrant careful planning and coordination in order to capture valuable resources for our respective research. As all these cyber technologies evolve around us, the basic problems pertaining to agricultural crops will still persist, only this time the solutions are more in-depth and can be solved at a faster pace since access to knowledge has become unlimited.

**FIGURE 4:**
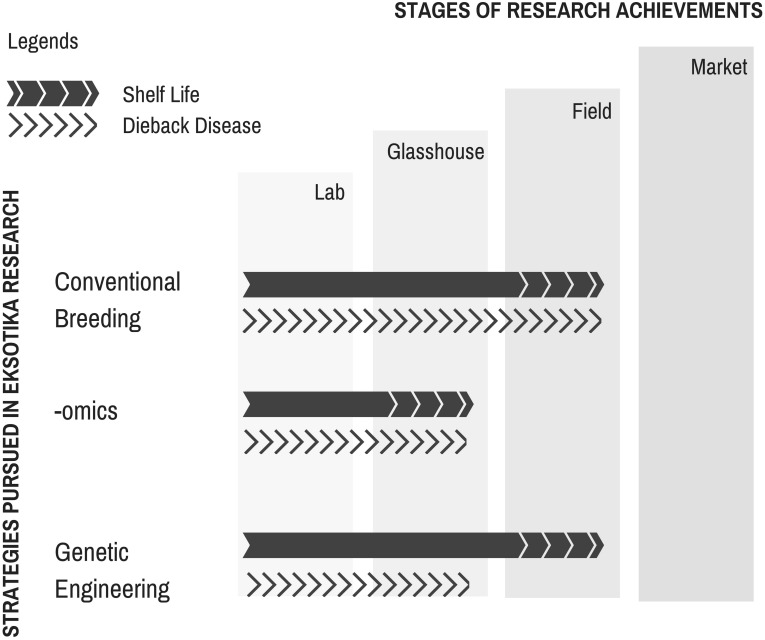
Summary of research work conducted on *Carica papaya* L. var. Eksotika in Malaysia.

The journey of Eksotika papaya research (summarized in **Figure 4**) is without doubt tough but still rewarding. The huge economic losses became an impetus and motivation for researchers to find a solution to sustain the papaya industry. Even though there is yet a long-term solution to the dieback problem, fundamental groundwork had been initiated with frontier technologies incorporated in short-, mid-, and long-term strategies by different research groups to address the challenge. The availability of the -omics databases (genomics, transcriptomics, proteomics, and metabolomics) will ultimately make it feasible to tackle the challenges more effectively by providing a more comprehensive data sets to gain novel insights into the principles of biological systems. It is with great hope, though, that such frontier technologies will be made cheaper in future to allow a wider coverage of the subject matter under scrutiny.

Research to extend the shelf-life of Eksotika fruit had, however, met with commendable progress using genetic engineering (GE) technology. GE technology has a huge potential to ensure a continuous supply of food. However, public acceptance of genetically modified (GM) crop has two-faced values (positive and negative values) despite the fact that there is no clear-cut evidence implicating current marketed GM crops have more health-related risks than conventionally bred crops. Reflecting this on GM Eksotika papaya hitting the oversea market indeed projects a very gloomy future if such viewpoints still persist with GM crops. It is a challenging path to venture, and it must be tread with caution. Hopefully with the comprehensive data sets provided by frontier -omics technologies, this will help to provide better crop safety assessments that can allay the public fears and convince them to accept GM crops with confidence. To obtain public confidence and acceptance, information on the GM technology and its potentials should be disseminated to the public and at a frequent occurrence through awareness programs. It is hopeful that such awareness programs can be made easier now as we are into a window era (with borderless spheres) and facing a screen for information is a norm for all generations. With an enhanced awareness and understanding on GM and GM-related products, this might cascade down to better acceptance of the GM products when they are released into the market.

Eksotika papaya has a bright future. It has gained a lot of attention ever since it was bred with good eating and aesthetic qualities for commercialization. An enormous amount of effort had been showered on sustaining it despite the threat of the fast spreading dieback disease, and limited capacity for distant market economy due to its short shelf-life fruit. Nevertheless, until another variety can surpass its aesthetic and quality fruit characteristics, Eksotika will remain an important papaya variety with export value that can contribute significantly to the world papaya varieties and Malaysian economy.

## Author’s Note

RS, MH, and RR registered their graduate studies at Universiti Putra Malaysia, and carried out major parts of their research work at the Malaysian Agricultural Research and Development Institute (MARDI), Serdang, Selangor, Malaysia. Hamid (2017) is a thesis material by the author of this review. The full thesis is not accessible online as it is in line with the author’s university policy. However, after a 2-year embargo, the partial content can be accessed online via http://www.psasir.upm.edu.my/ as well as a printed version of the thesis can be made by request to the university.

## Author Contributions

All authors listed have made equal intellectual contribution to the work and approved it for publication.

## Conflict of Interest Statement

The authors declare that the research was conducted in the absence of any commercial or financial relationships that could be construed as a potential conflict of interest.

## Footnotes

1 www.arabidopsis.org

2 http://blast.jcvi.org/euk-blast/plantta_blast.cgi

3 www.genome.jp/kegg

4 www.phytozome.net
